# Integrating Large Language Models Into Trauma Education for Medical Students: Randomized Controlled Pilot Trial

**DOI:** 10.2196/79134

**Published:** 2026-03-17

**Authors:** Joona Gustafsson, Erno Lehtonen-Smeds, Niklas Pakkasjärvi

**Affiliations:** 1 Wellbeing Services County of Southwest Finland, University of Turku, PO Box 52, Turku, 20521, Finland; 2 Wellbeing Services County of Ostrobotnia, Vaasa Central Hospital, Vaasa, Finland

**Keywords:** large language models, LLM, medical education, artificial intelligence, ChatGPT, medical students, teamwork, decision-making

## Abstract

**Background:**

The exponential growth of medical knowledge presents a paradox for modern medical education. While access to information is immediate, applying it in a clinically meaningful way remains a challenge. Large language models (LLMs), such as ChatGPT, are widely used for information retrieval, yet their role in dynamic, high-pressure clinical learning remains poorly understood.

**Objective:**

This study aims to evaluate whether unstructured access to an LLM improves decision-making, teamwork, and confidence in trauma education for medical students.

**Methods:**

This randomized controlled pilot study involved 41 final-year medical students participating in a trauma simulation session. Students self-selected into teams of 4 to 6 and were randomized to either an LLM-assisted group (ChatGPT-4o mini) or a control group without LLM access. All teams completed 18 video-based trauma scenarios requiring time-sensitive clinical decisions. Prompting was unrestricted. Confidence and trauma exposure were assessed using pre- or postquestionnaires. Facilitators rated teamwork (1-5), decision accuracy, and response times. Knowledge retention was measured 4 weeks later via an online quiz.

**Results:**

Confidence in trauma management improved in both groups (*P*<.001), with larger gains in the non-LLM group (*P*=.02). LLM support did not enhance the decision accuracy or speed and was associated with longer response times in some complex cases. Teams without LLMs demonstrated more active discussion and scored higher in teamwork ratings (median 5.0 [IQR 5.0-5.0] vs median 3.5 [IQR 3.0-4.5]; *P*=.08). Students primarily used the LLM for fact-checking but reported vague or overly general responses. Knowledge retention was high across both groups and did not differ significantly (*P*=.33).

**Conclusions:**

While students appreciated the inclusion of artificial intelligence (AI), unstructured LLM use did not improve performance and may have disrupted the group reasoning. The use of non-English prompting likely contributed to lower AI performance, underscoring the importance of language alignment in LLM applications. This pilot study highlights the need for structured AI integration and targeted instruction in AI literacy. Simulation-based trauma education proved effective and well received, but optimizing the educational value of LLMs will require thoughtful curricular design. Further studies with more students are needed to define best practices for LLM use in clinical education.

## Introduction

The exponential growth of medical knowledge presents a paradox for modern medical education. While access to information is more immediate than ever, the ability to filter, contextualize, and apply that information remains a significant challenge. This concern is particularly relevant for medical students, who often lack the clinical experience to distinguish critical insights from background data  [1,2]. Thus, a growing need for tools that provide information and help organize and synthesize it in clinically meaningful ways is evident.

Large language models (LLMs) such as ChatGPT have emerged as promising tools in this context  [3]. Initially developed for language processing, LLMs operate on a probabilistic framework that predicts the next word in a sequence based on patterns in their training data  [4]. Though not originally designed as knowledge engines, these models are increasingly used to retrieve and summarize medical information. LLMs can assist with fact-checking, clinical guidance, and even reflective reasoning when prompted effectively  [3,5,6]. However, their outputs must be interpreted cautiously, as they lack proper understanding and may generate plausible but incorrect information [7].

LLMs have excelled in diagnostic tasks under controlled conditions  [8], yet these results may not generalize to dynamic clinical environments where uncertainty, time pressure, and teamwork play critical roles  [9].

Despite growing enthusiasm for integrating artificial intelligence (AI) into medical education, there is limited high-quality evidence of its use in interactive, team-based, or high-stakes clinical learning settings. A systematic review by Lucas et al [5] highlighted the potential of LLMs to support learning but noted a lack of empirical trials in real-time clinical scenarios. Similarly, Nguyen [7] raised concerns about automation bias, where overreliance on AI can impair critical reasoning. To our knowledge, no randomized studies have directly examined how unstructured LLM support affects team dynamics and decision-making accuracy during trauma simulations.

To address this gap, we conducted a randomized controlled pilot study to examine whether a large language model (ChatGPT) can be meaningfully integrated into trauma simulations, how students use it in practice, and what problems emerge from its unstructured application. Students received no prior training in prompt formulation or AI use and were free to interact with the model at their discretion, reflecting real-world conditions. The study focused on observable outcomes including confidence, facilitator-rated teamwork interactions, decision accuracy, response times, and knowledge retention. These measures were used to characterize general patterns of LLM use within the constraints of the simulation setting. The aim was to generate evidence on how unstructured LLM availability may affect the learning process in team-based clinical simulations and to inform the design of future hypothesis-driven studies on structured AI integration in medical education.

## Methods

### Study Framework

Simulation-based trauma education relies on active, team-based learning, where decision-making under time pressure and effective communication are central to performance. These principles guided our choice of outcomes, focusing on confidence, facilitator-rated teamwork interactions, decision accuracy, response times, and short-term knowledge retention.

Introducing an LLM into this setting relates to emerging concepts in human-AI interaction, such as cognitive offloading and automation bias, which may influence how teams share information and make decisions. In the lack of consensus frameworks for integrating LLMs into team-based clinical simulations, we adopted a pragmatic, performance-focused approach to examine how students chose to use the LLM and whether its presence influenced observable aspects of the learning process.

This framework informed the design of this pilot study, which was not intended as an implementation trial but as an exploratory evaluation of how unstructured LLM availability may affect learning and team performance in trauma simulation.

### Study Design and Setting

This single-center, exploratory pilot randomized controlled trial was conducted between January 13, 2025, and April 15, 2025, as part of the final-year trauma training curriculum at Vaasa Central Hospital, University of Turku, Finland. The study aimed to examine whether an LLM (ChatGPT) can be meaningfully integrated into trauma simulations, how students use it in practice, and what challenges arise from its unstructured application. The trial followed general CONSORT (Consolidated Standards of Reporting Trials) guidelines (Checklist 1) for randomized educational interventions.

### Ethical Considerations

The study was reviewed by the Clinical Research Center, VARHA (Wellbeing Services County of Southwest Finland), and deemed exempt from formal ethical approval in accordance with institutional and national regulations governing educational research involving adult participants. The procedures followed were consistent with institutional guidelines and the principles of the Declaration of Helsinki.

All participating students received written and verbal information about the study and provided informed consent prior to participation. Participation was voluntary, and students were informed that declining participation would not affect their course standing or academic evaluation. No financial compensation or other incentives were provided.

No patient data were used in this study. Students accessed the LLM through a publicly available interface without logging in, and no identifiable student information was entered into the model at any point. Interactions with the LLM were not stored or exported, and no prompts or outputs were retained by the research team.

Only deidentified team-level performance data were collected. Individual-level responses were anonymized prior to analysis, and no identifiable information was reported. Data are available upon reasonable request. The study was preregistered on the Open Science Framework [10] on April 15, 2025. As with any generative AI tool, there is potential for language-related bias or factual inaccuracies, particularly when prompting in a language other than English. These considerations are discussed as limitations of the intervention.

### Participants and Randomization

A total of 41 final-year medical students from the University of Turku, a publicly funded institution with a standardized curriculum, participated in the study. All students were enrolled in a voluntary trauma care simulation course. Students had previously self-selected into teams of 4 to 6 individuals at the beginning of the academic year, and these groups were preserved for the study. All participants had prior, curriculum-based simulation training experience.

A total of 8 pre-existing student teams (clusters) were randomized 1:1, resulting in 4 teams allocated to the LLM group (using ChatGPT-4o mini as a supportive tool during simulations) and 4 teams allocated to the control group (no LLM support). All scenario-level outcomes (decision accuracy, response time, and teamwork scores) were aggregated to the team level for analysis to avoid pseudoreplication. A computer-generated randomization sequence was created by a researcher not involved in the simulation sessions. Group assignments were communicated to the facilitator immediately prior to each session. Due to the visible presence of the laptop used for LLM access, facilitators were not blinded to group allocation.

Because students had pre-established simulation groups, randomization was performed at the team level. Each intact team was assigned as a single unit to either the LLM or control condition. As the intervention was delivered to teams and all scenario-based decisions were made collectively, the team constituted the unit of randomization.

### Intervention (LLM use)

Teams assigned to the intervention group were provided access to ChatGPT-4o mini via the publicly available free web interface. The same model was used in all sessions, with no premium features, plug-ins, or browsing capabilities enabled.

No prompting guidelines or technical instructions were given, and the interface remained unconfigured. Participants were free to interact with the model at their discretion and in any language; in practice, all teams chose Finnish. This decision to allow unstructured use was intentional, aiming to capture how students would naturally engage with an unconfigured, publicly available LLM in a clinical learning environment. Participants’ prior familiarity with LLMs stemmed solely from personal experience, as no formal AI literacy or prompt engineering instruction was part of the curriculum.

All LLM-assisted sessions took place between January 13, 2025, and April 15, 2025. The model label displayed in the interface during all sessions was “ChatGPT-4o mini.” Prompts and outputs were not systematically logged, as the study was designed to observe naturalistic use rather than evaluate prompt formulation. Students generally entered short, broad factual questions; often definitions, procedural steps, or indications, and invariably in Finnish.

### Simulation Protocol

The instructional environment combined video-based scenario introductions with hands-on simulation using mannequins and procedural models (Multimedia Appendix 1). Each scenario began with a short video vignette sourced from publicly available YouTube material, used only to establish the clinical context and create a sense of urgency. All scenario content, tasks, and questions used in the simulation were developed by the study team. Students then managed each case using a human-like mannequin or low-fidelity simulator, performing the relevant assessments and interventions.

For example, pneumothorax scenarios included interpretation of simulated X-rays, verbal explanation of chest tube placement steps, and the procedure itself on a dedicated cardboard model. Throughout each scenario, students were asked open or multiple-choice questions related to diagnostics and management. Participants in the intervention arm could consult the LLM at any point for factual support or practical guidance. All scenarios were presented in the same order for all teams. Feedback was provided by trained facilitators after each case.

### Data Collection and Outcome Measures

Data were collected before, during, and after the simulation sessions. Baseline characteristics, including self-reported trauma exposure and pre-session confidence ratings, are summarized in Table 1. Trauma exposure was categorized as curriculum-based (grade 1) or more extensive (grades 2‐3) based on students’ reported prior experience.

**Table 1.** Baseline characteristics of participating students (N=41).

Characteristics	LLM^a^ group (n=22)	Control group (n=19)	*P* value^b^
Baseline confidence score, median (IQR)	2.17 (1.75‐2.67)	2.33 (2.00‐2.83)	.35
Prior simulation experience, n (%)	22 (100)	19 (100)	—^c^
Prior trauma exposure, n (%)^d^			
Grade 1	16 (73)	12 (63)	.51
Grade 2‐3	6 (27)	7 (37)	.74

a LLM: large language model.

b *P* values were calculated using Mann–Whitney *U* test for continuous variables and Fisher exact test for categorical variables.

c Not applicable.

d Trauma exposure was categorized as grade 1 (curriculum-based) or grades 2‐3 (extended experience).

Teamwork collaboration was assessed in real time by the session facilitator using a simple 1 to 5 rating scale developed for the trauma curriculum. Scores reflected observable indicators of collaboration, including verbal participation by multiple team members, shared decision-making, turn-taking, and the degree of coordinated problem-solving during each scenario. The tool was designed to provide a practical measure of team interaction in this pilot setting and was not intended as a validated teamwork instrument. Because the presence of the LLM interface was visible, blinding of outcome assessors was not feasible, and the potential for detection bias is acknowledged. Facilitators also recorded decision accuracy (correct or incorrect) and time to decision during each scenario. Following the simulation, participants in the LLM group completed a short, bespoke postsession questionnaire developed for this pilot study. The instrument included 14 statements rated on a 1 to 5 Likert scale (1=“strongly disagree,” 5=“strongly agree”) covering perceived usefulness, ease of use, impact on learning, confidence, perceived limitations, and attitudes toward future integration. Three additional open-ended questions asked students to describe which aspects of the LLM were most helpful, what challenges they encountered, and how the tool could be improved for future training. The questionnaire was designed to obtain exploratory feedback rather than generate validated outcome measures, and responses were summarized descriptively to identify common patterns in students’ perceptions. Knowledge retention was assessed via an anonymous online quiz 4 weeks after the simulation using a short, scenario-aligned online quiz. The quiz consisted of 7 multiple-choice questions, each with 4 answer options and 1 correct response. Items were blueprinted directly to the clinical content of the simulation scenarios and covered core trauma competencies, including chest drain placement, pleural puncture technique, lower-leg compartment anatomy, initial management of pelvic trauma, recognition of tension pneumothorax, indications for computed tomography imaging in blunt head trauma, and cervical spine precautions after high-energy mechanisms. Each correct answer contributed 1 point for a total possible score of 7, and results were analyzed as the proportion of correct responses. The quiz was designed for formative assessment; no formal reliability analysis was performed due to the small number of items and the pilot nature of the study.

### Statistical Analysis

All primary team-based performance outcomes were analyzed at the team (cluster) level, corresponding to the unit of randomization. For each team, mean values across all 18 scenarios were calculated for response time, facilitator-rated teamwork collaboration interactions, and decision accuracy (correct or incorrect). Scenario-level comparisons (LLM vs control) for these team-level outcomes were conducted using independent-samples 2-tailed *t* tests or Mann-Whitney *U* tests, depending on distributional assumptions. Effect sizes (Cohen *d* or rank-biserial correlation) were reported where appropriate, and normality was assessed with the Shapiro-Wilk test.

Individual-level outcomes, including confidence ratings, trauma exposure, and knowledge-retention scores were collected at the participant level and summarized descriptively. These measures were not used for between-group inferential testing and were not cluster adjusted. A 2-sided *P*<.05 was considered statistically significant for team-level comparisons. As this was an exploratory pilot study, no primary end point was prespecified and no formal power calculation was performed; all eligible students who volunteered were included.

## Results

**Figure 1.**
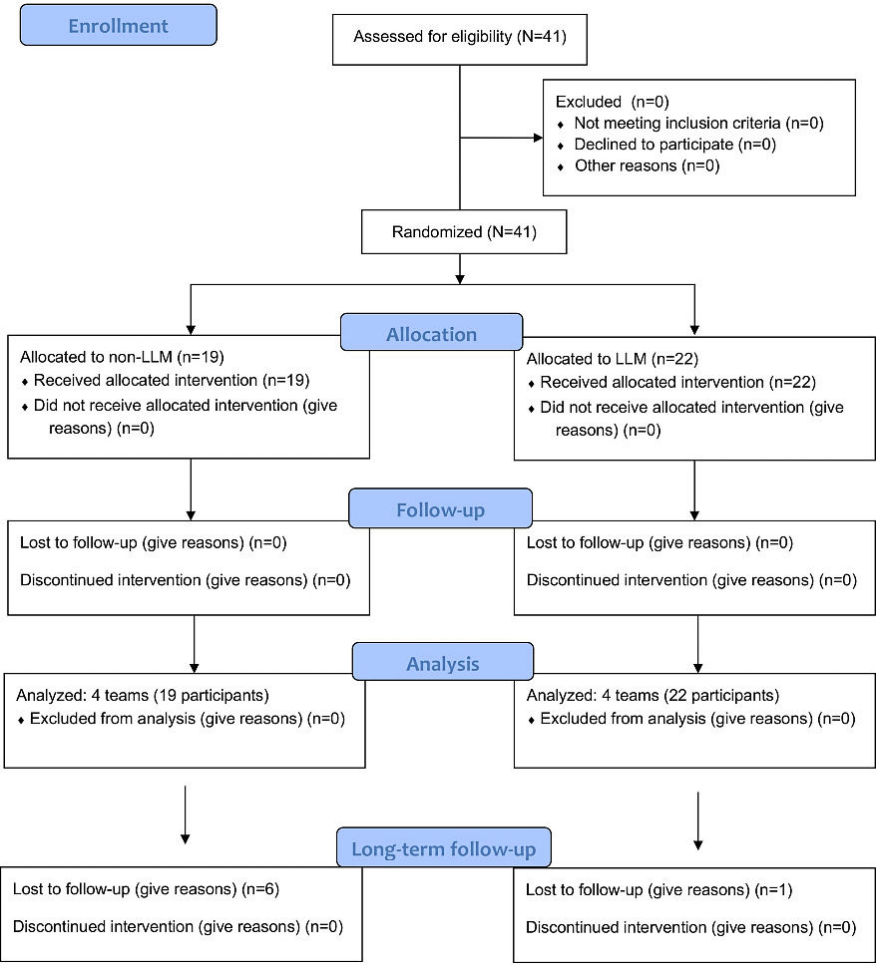
CONSORT (Consolidated Standards of Reporting Trials) flow diagram. LLM: large language model.

A total of 41 students participated in the study, forming the pre-existing teams that constituted the unit of randomization (Figure 1). Baseline characteristics, including initial confidence scores and prior trauma exposure, were similar between groups (Table 1).

Analyses were conducted at the team level, corresponding to the unit of randomization. Across all participants, self-reported confidence in trauma management increased significantly following the simulation session overall (*P*=1.3 × 10^–^⁸; Table 2).

When comparing randomized teams, the non-LLM group demonstrated a greater median improvement in self-reported confidence compared with the LLM-assisted group (*P*=.02).

**Table 2.** Pre- and postsession confidence^a^ scores (1–5) in trauma management.

Confidence domain^a^ and trauma experience		Presession, median (IQR)	Postsession, median (IQR)	*P* value^b^
Treatment				
Curriculum-based		1.5 (1.0‐2.0)	2.0 (2.0‐3.0)	<.001
More extensive		2.0 (1.0‐3.0)	3.0 (2.0‐3.0)	.04
Decision-making				
Curriculum-based		3.0 (2.0‐3.0)	3.0 (3.0‐3.0)	<.001
More extensive		3.0 (3.0‐3.0)	3.0 (3.0‐3.0)	.99

a Confidence was assessed in two domains: (1) recognizing and treating life-threatening trauma situations (eg, hemodynamic instability, and pneumothorax) and (2) making decisions about patient disposition (eg, referral to specialized care, lower-level center, or discharge).

b Statistical significance was determined using the Wilcoxon signed-rank test, which can detect paired changes even when median values appear unchanged.

No statistically significant differences were observed between groups in mean decision-making accuracy (*P*=.06; *r*=0.75) or mean response time (*P*=.88; d=0.11; Table 3). Teamwork performance (5, 5, 5, 5 for non-LLM teams and 3, 3, 4, 5 for LLM-assisted teams) did not differ significantly between groups (*P*=.08; d=–1.85), although non-LLM teams exhibited higher median collaboration scores (5.0 vs 3.8). A trend toward longer response times in several complex scenarios persisted among LLM-assisted teams (Table 3). Neither cohort achieved consistently correct responses across all scenarios. Scenario-specific accuracy for both groups is presented in Table 3. Facilitator observations indicated that LLM-assisted teams tended to verbalize less, with several scenarios characterized by students turning to the laptop or silently reviewing the model’s output instead of engaging in group discussion. In contrast, non-LLM teams typically talked through the cases more actively. Despite reduced interaction in the LLM groups, overall response times were not consistently longer, likely because consultation with the LLM often replaced, rather than added to, internal discussion time.

**Table 3.** Scenario-specific response times and accuracy^a^.

Scenario	Expected action	Non-LLM^b^ (s), median (IQR)	LLM (s), median (IQR)	*P* value^c^	Accuracy non-LLM/LLM (%)^d^
1	Recognize and manage a compromised airway	13.0 (11.0‐18.0)	17.0 (14.5‐18.75)	.88	100/100
2	Demonstrate correct placement and attachment of a chest tube	633.0 (499.0‐718.5)	640.5 (544.75‐697.5)	.99	100/100
3	Identify appropriate treatment options for tibial fractures	47.0 (38.75‐58.25)	42.0 (34.75‐49.75)	.89	100/100
4	Classify an open fracture	16.0 (5.5‐30.25)	9.5 (3.5‐36.5)	.99	100/100
5	Explain the classification of open fractures	24.5 (15.5‐41.25)	55.5 (41.75‐69.5)	.69	100/100
6	Identify the compartments of the lower leg	12.0 (8.5‐15.25)	29.0 (21.5‐41.5)	.11	100/100
7	List the anatomical components in each lower leg compartment	13.5 (10.0‐28.75)	20.0 (13.5‐27.0)	.89	50/100
8	Describe the diagnostic steps for compartment syndrome	188.5 (164.25‐203.5)	135.5 (102.75‐155.75)	.11	100/100
9	Describe the intramedullary tibial nailing procedure	45.0 (42.5‐62.5)	44.5 (36.5‐56.25)	.72	75/0
10	Identify and rank common complications associated with tibial nailing	82.5 (51.75‐112.75)	133.0 (74.25‐183.75)	.69	50/0
11	Determine discharge criteria based on trauma mechanism	221.0 (176.0‐234.0)	129.0 (97.0‐140.25)	.20	100/100
12	Outline the key steps in trauma patient evaluation	17.0 (16.5‐19.5)	11.0 (10.0‐14.25)	.19	100/100
13	Recognize pelvic fracture, assess for hemorrhage, and determine stabilization strategy	20.0 (15.0‐46.5)	42.0 (20.0‐64.75)	.89	100/100
14	Describe the management steps for massive bleeding in pelvic fractures	36.5 (29.25‐40.25)	57.0 (41.0‐67.25)	.34	100/100
15	Identify and manage a tension pneumothorax	12.5 (6.5‐17.0)	15.5 (10.75‐21.25)	.99	100/100
16	Identify indications for thoracotomy after chest drain insertion	36.0 (24.0‐41.5)	25.0 (17.75‐35.5)	.86	100/0
17	Explain the limitations of imaging in blunt abdominal trauma	11.5 (10.5‐30.75)	26.5 (12.75‐46.5)	.89	100/100
18	Identify indications for CT^e^ imaging in blunt head trauma	45.5 (14.75‐78.0)	10.5 (7.5‐18.25)	.20	100/100

a Median (IQR) values are presented in seconds.

b LLM: large language model.

c *P* values were calculated using Mann-Whitney *U* tests.

d Accuracy is reported as the percentage of teams with a correct response (correct teams/total teams).

e CT: computed tomography.

Observational notes documented that teams with LLM access often deferred decisions to the AI tool without sufficient internal discussion or verification. Participants in the LLM-assisted teams completed a structured postsession questionnaire consisting of 14 Likert-scale items and three open-ended questions. Median Likert ratings indicated neutral to mildly positive perceptions of overall usefulness (median 3, IQR 3‐4) and impact on learning (median 3, IQR 2‐3), while ease of use showed mixed responses (median 2‐4 across items). Limitations were commonly endorsed, particularly regarding inaccuracies when prompting in Finnish (median 4, IQR 3‐4). Despite these challenges, students expressed generally positive attitudes toward future integration of LLMs into medical education (median 4, IQR 3‐4). Themes from open-ended comments reflected (1) primary use of the LLM for fact-checking and procedural clarification, (2) frustration with vague or overly general answers, and (3) support for more structured or guided integration in future sessions. Full item-level results are summarized in Table 4. Several factual inaccuracies were identified when the model was prompted in Finnish.

**Table 4.** Postsession questionnaire results large language models (LLMs; LLM-assisted teams).

Item	Domain	Median (IQR)	
1. LLM improved ability to manage trauma cases	Usefulness	3 (3‐4)	
2. LLM provided valuable insights for decision-making	Usefulness	3 (3‐4)	
3. LLM was simple and intuitive to use	Ease of use	2 (2‐3)	
4. I felt comfortable using the LLM during the simulation	Ease of use	4 (3‐4)	
5. LLM had a positive effect on my learning experience	Learning impact	3 (2‐3)	
6. LLM helped me better understand trauma care principles	Learning impact	3 (3‐4)	
7. I am satisfied that LLM support was included	Satisfaction	3 (3‐3)	
8. I would recommend including LLM support in future training	Satisfaction	4 (3‐4)	
9. LLM increased my confidence in handling real trauma cases	Confidence	2 (2‐3)	
10. I feel better prepared to make clinical decisions after using the LLM	Confidence	3 (2‐4)	
11. At times, LLM support was more harmful than helpful	Limitations	3 (3‐3)	
12. I encountered inaccuracies or limitations in LLM responses	Limitations	4 (3‐4)	
13. LLMs should be integrated into medical education	Future integration	4 (3‐4)	
14. I am willing to use LLM support in future training	Future integration	4 (4‐4)	

A total of 34 out of 41 students completed the knowledge retention assessment 4 weeks post session. The proportion of correct responses did not differ significantly between groups (non-LLM group: 89.2%; LLM group: 84.4%; *P*=.33).

## Discussion

### Principal Findings

In this exploratory randomized pilot study, unstructured access to an LLM did not improve decision-making speed or accuracy in trauma simulations. Scenario-specific patterns were observed: in 3 of the 18 scenarios, all LLM-assisted teams selected incorrect answers, whereas non-LLM teams showed variable but generally higher accuracy. Teams using the LLM demonstrated lower teamwork scores and were less interactive, frequently deferring decisions to the AI tool. Students in both groups reported increased confidence after the session, without additional benefit from LLM use. Observational notes suggest that poor prompt formulation and overreliance on the model may have contributed to delays and decision errors. However, prompt precision was not formally measured and should be addressed in future studies. Several AI outputs appeared to contain factual inaccuracies, particularly when prompted in Finnish; these were not systematically evaluated and are therefore discussed only as qualitative observations to guide future research.

Facilitator observations provided additional insight into how LLM access influenced team behavior. Teams with LLM support frequently paused to consult the model or read its output, which appeared to replace some of the natural verbal processing that typically occurs during team-based simulations. This shift contributed to lower teamwork ratings despite comparable response times. The LLM often produced rapid but broad or nonspecific answers; as a result, consultation rarely prolonged task completion but did interrupt collaborative reasoning. These behavioral patterns highlight the importance of structured guidance for LLM use to prevent disruption of team communication in fast-paced clinical environments.

### Comparison With Prior Literature

These findings align with emerging literature on AI in medical education. LLMs such as ChatGPT offer promising support for learners but may struggle to deliver context-rich reasoning or reliably accurate responses without structured prompts or domain tuning [5,7,11]. Prompting quality has emerged as a key determinant of model performance. Recent studies emphasize that targeted prompt engineering can significantly improve output accuracy, relevance, and response time, particularly in procedural and diagnostic contexts [12-14]. This supports the notion that structured AI literacy or microtraining could mitigate some of the issues observed during real-time trauma simulations.

Moreover, the multilingual context matters: LLMs trained primarily in English may underperform in other languages, underscoring the need for language-aligned or locally fine-tuned models to ensure equitable educational outcomes across linguistic contexts [15,16]. Few studies have examined the impact of LLMs on team-based, high-stakes simulation environments. Evidence from trauma team simulations shows that context can strongly influence teamwork and cognitive load even without AI, and human-AI studies warn that automation bias and AI teammate behaviors can disrupt coordination if not carefully structured [17-21]. These factors may help explain the neutral or negative performance effects observed in our trial.

### Implications of Findings

This study highlights how unstructured use of LLMs may influence clinical reasoning and teamwork during time-sensitive scenarios. Reliance on AI tools can inadvertently disrupt the interactive human problem–solving processes that are critical to trauma care. These behavioral effects emphasize the need to integrate LLMs into medical education in a structured and guided manner, rather than assuming benefit from mere access. This aligns with broader observations in surgical education, where nontechnical competencies such as teamwork, communication, and decision-making are increasingly recognized as critical learning objectives but often underemphasized in training frameworks [22]. Although not a primary outcome, several factual inaccuracies were observed in AI-generated outputs during the simulations. Students prompted the model in Finnish, which likely contributed to these errors, as most LLMs are trained primarily on English-language data. These findings underscore the importance of improving multilingual performance and localization in LLMs to ensure applicability across diverse health care systems [15]. Beyond immediate simulation performance, a broader concern is the potential shift in learning behaviors among students [23]. Overreliance on AI tools may reduce active engagement with primary sources and critical appraisal skills—competencies essential for clinical reasoning and lifelong learning. Educators should therefore emphasize structured AI literacy and clear pedagogical frameworks to ensure that LLMs function as supportive decision aids rather than substitutes for clinical judgment [7].

### Strengths and Limitations

Limitations of this study include the relatively small sample size and discretionary use of LLMs, which may have introduced variability in engagement. Students self-selected into teams prior to randomization, potentially affecting group dynamics. Knowledge retention was generally high across both groups, but detailed instructor-led debriefings of all cases may have minimized group differences, making comparisons less meaningful. Additionally, no formal power calculations were performed; all eligible students who volunteered were included, and no participants declined.

A key limitation concerns the use of nonvalidated instruments for teamwork and decision accuracy, reflecting the pilot nature of the study. Importantly, students received no prompting or language-use instruction, and all teams independently chose to interact with the LLM in Finnish. This unstructured use may have contributed to variable prompting quality, factual inaccuracies, and longer response times due to language mismatch with the model. At the same time, this unstructured and naturalistic use of the tool is also a strength of the study, as it mirrors how students without prior AI training are likely to engage with LLMs in real-world settings. This highlights the need for structured prompting instruction and language-aligned models in future educational interventions and provides a useful baseline against which to design and evaluate more structured trials.

### Future Directions

Further research should explore the role of AI in structured learning environments, emphasizing support for, rather than replacement of, active clinical reasoning. This pilot study underscores both opportunities and limitations of integrating LLMs into clinical training. It provides a foundation for refining educational strategies, and future studies with greater statistical power will help define the optimal use of these tools in medical education. Ultimately, the question is not whether to use AI in medical education, but how to implement it effectively to support human reasoning and teamwork.

## Supplementary material

10.2196/79134 Multimedia Appendix 1 Detailed description of simulation setup.

10.2196/79134 Checklist 1 CONSORT checklist.

## Abbreviations

AI: artificial intelligence
CONSORT: Consolidated Standards of Reporting Trials
LLM: large language model
