# Supplementary material for: Integrating Large Language Models Into Trauma Education for Medical Students: Randomized Controlled Pilot Trial
Source: JMIR Med Educ. 2026 Mar 17;12:e79134. doi: 10.2196/79134 (PMC12994756; doi:10.2196/79134)
Supplement: Multimedia Appendix 1 [file mededu-v12-e79134-s001.docx]

**Appendix 1. Structure of the Simulation-Based Trauma Training**

The trauma simulation session was designed to combine immersive case-based learning with practical skill development. Each session included the following structured components:

1. **Scenario Introduction via Video Vignettes**
   - Each of the 18 trauma scenarios began with a short video clip (30–90 seconds) to establish clinical urgency and context.
   - Videos were selected from publicly available sources, including medical documentaries and dramatizations (e.g., hospital series), and edited to focus on relevant symptoms and cues.
2. **Case Management with Simulation Mannequins or Models**
   - After viewing the vignette, student teams interacted with a human-like mannequin or procedural model representing the trauma patient.
   - Cases included conditions such as pneumothorax, compartment syndrome, long bone fractures, and airway obstruction.
   - Students were tasked with assessing the simulated patient, identifying life-threatening issues, and determining an appropriate management plan.
3. **Procedural Task Component**
   - Each scenario included a hands-on task using low-fidelity models. For example:
     - **Pneumothorax:** Interpretation of a simulated X-ray, verbal description of chest tube placement steps, and performance of the procedure on a cardboard thorax model.
     - **Fractures and immobilization:** Use of splints and braces on limb models.
     - **Airway scenarios:** Use of mannequins for jaw thrusts, airway opening, and assessment.
4. **Question-Based Decision-Making**
   - Teams were intermittently presented with open-ended or multiple-choice questions during the case to assess clinical reasoning and reinforce decision points.
   - Responses were recorded and used as part of the team’s performance assessment.
5. **LLM Access in the Intervention Group**
   - Students randomized to the LLM-assisted group were free to consult the large language model (ChatGPT) at any point during the case for fact-checking, differential diagnoses, or procedural guidance.
   - Prompts were not standardized. All students chose to interact with the model in Finnish.
6. **Structured Debriefing**
   - Each case concluded with a short facilitator-led debrief, focusing on clinical priorities, decision quality, and teamwork.
   - Procedural feedback was also provided based on task performance.

This multi-modal approach was chosen to reflect realistic clinical workflow, support active learning, and allow the evaluation of both individual reasoning and team interaction under pressure.

**Teamwork Assessment:**
Team performance was rated on a 1–5 scale by the facilitator following each scenario. The rating was based on observable team behaviors, including:

- clarity of communication,
- distribution of roles,
- shared decision-making,
- engagement of all team members.

This scale was not formally validated and was used pragmatically to capture clear differences in team dynamics. Due to the pilot nature of this study, validated tools were not implemented. Future studies will incorporate validated assessment tools to ensure standardization, inter-rater reliability, and psychometric rigor.

**Decision Accuracy Assessment:**
Each scenario included one or more predefined key clinical actions (e.g., initiating pleural decompression for a tension pneumothorax). These expected actions were developed by a panel of trauma educators prior to the sessions. Decision accuracy was defined by whether these critical actions were identified and acted upon. Most scenarios were structured to have binary (correct/incorrect) outcomes to minimize subjectivity. In scenarios with multiple acceptable responses, agreement was established based on current trauma guidelines and expert consensus.
